# Hybrid FDG-PET/MRI for Diagnosis and Clinical Management of Patients with Suspected Perihilar Cholangiocarcinoma: A Feasibility Pilot Study

**DOI:** 10.1007/s13139-024-00873-2

**Published:** 2024-08-01

**Authors:** D. M. de Jong, K. Chehin, T. L.N. Meijering, M. Segbers, L. M.J.W. van Driel, M. J. Bruno, B. Groot Koerkamp, J. N.M. IJzermans, F. A. Verburg, Q. G. de Lussanet de la Sabloniere, R. S. Dwarkasing

**Affiliations:** 1 Department of Gastroenterology and Hepatology, Erasmus MC University Medical Center, Rotterdam, the Netherlands; 2 Department of Radiology and Nuclear Medicine, Erasmus MC University Medical Center, Rotterdam, the Netherlands; 3 Department of Surgery, Erasmus MC University Medical Center, Rotterdam, the Netherlands

**Keywords:** Hilar Cholangiocarcinoma, PET/MRI, Clinical Decision Making, Biliary Tract cancer

## Abstract

**Purpose:**

Recently introduced hybrid 2-[18 F]-fluoro-2-deoxy-D-glucose (18 F-FDG) Positron Emission Tomography (PET) combined with Magnetic Resonance Imaging (MRI) may aid in proper diagnosis and staging of perihilar cholangiocarcinoma (pCCA). The aim of this study is to assess the effect of 18 F-FDG PET/MRI on diagnosis and clinical decision making in the pre-operative work up of pCCA.

**Methods:**

In this single-centre pilot study patients with presumed resectable pCCA underwent state-of-the-art 18 F-FDG hybrid PET/MRI using digital silicone photomultiplier detectors integrated within a 3-Tesla bore. Data were collected on several baseline and imaging characteristics. The primary outcome measure was the added diagnostic information and the effect on clinical decision making. Secondary aim was to correlate quantitative PET signal intensity to patient- and tumour characteristics. High and low SUVmax subgroups related to the mean value were made. Significance of lesion- and patient characteristics with the high and low SUVmax subgroups, as well as TLR and TBR, was evaluated with Fisher’s exact test or Mann-Whitney-U test.

**Results:**

In total 14 patients were included (mean age 62.4 years, 64% male). Final diagnosis was pCCA in 10 patients (71.4%), follicular lymphoma in one patient (7.1%) and benign disease in the remaining three patients. FDG-PET/MRI added valuable diagnostic information in six (43%) patients and affected clinical decision making in two of these patients (14%) by increasing confidence for malignancy which lead to the decision for surgery on short term. High SUVmax values were seen in half of cases with pCCA and half of cases with non-cancerous lesions. In addition, high SUVmax values were directly associated with primary sclerosing cholangitis when present (*p* = 0.03).

**Conclusion:**

Simultaneous 18 F-FDG-PET/MRI added diagnostic information in six of fourteen patients and influenced clinical decision making in two patients (14%) with presumed resectable pCCA.

## Introduction

Perihilar cholangiocarcinoma (pCCA) is an uncommon malignancy and often patients present at a late stage with advanced disease [1]. Radical surgical resection or liver transplantation are the only potential means of achieving long term survival [2, 3]. Many patients have locally advanced disease with vascular encasement, positive regional or extraregional lymph nodes, or distant metastasis upon first presentation [4]. Additionally, up to 47% of the eligible patients are found to have unresectable disease during explorative laparotomy [3, 5, 6].

In current clinical practice, computed tomography (CT) and magnetic resonance imaging (MRI) are used to evaluate local tumour extent and resectability of pCCA. CT with intravenous contrast is especially useful for determining tumour extent and in particular vascular involvement of the hepatic artery and portal vein, including distant metastases [7]. MRI in combination with cholangiography (MRCP) provides detailed anatomical information of the biliary tree, which is important for surgical planning due to a wide variety of anatomical variations [8, 9]. Both CT and MRI/MRCP understage resectable pCCA often, as underlined by the high number of patients with unresectable disease at explorative laparotomy [3, 5, 6]. Pang et al. demonstrated the value of dual-time point 18 F-fluorodeoxyglucose (FDG) positron emission tomography (PET) CT (PET/CT) for primary tumour location(s), including lymph node metastases in pCCA. In addition, the authors described that the maximum standardized uptake value (SUVmax) may be indicative for tumour aggressiveness [10]. The last years, more evidence has been published on the incorporation of 2-[18 F]-fluoro-2-deoxy-D-glucose (18 F-FDG) PET imaging into the current standard of care for biliary tract cancer staging, but the exact role remains controversial [11]. Primarily PET/CT has not been the major breakthrough as hoped.

Recently, 18 F-FDG PET with MRI (PET/MRI) has become available. FDG-PET/MRI has shown additional value for pre-treatment work up in several malignancies, such as breast- and pancreatic cancer [12, 13]. The combination of the information on soft-tissues obtained by the MRI and metabolic information of the PET could prove a favourable utility. So far, small studies have been performed in patients with hepatobiliary neoplasms, including pCCA. It was reported that simultaneous hybrid FDG-PET/MRI has promising benefits over conventional preoperative imaging with CT and MRI/MRCP or combined with PET/CT [14–17]. In the study of Obmann et al. on sixteen patients with hepatobiliary neoplasms, FDG-PET/MRI correctly changed the cTNM stage in 22% of patients with consequent change in management in 11% of patients with extrahepatic cholangiocarcinoma [14].

When measuring SUV in addition to visual assessment of PET images one should consider possible confounding effects owing to differences among PET systems and differences in acquisition and image reconstruction techniques that may substantially affect the measured SUV values [18]. For these reasons efforts are made to harmonize and standardize SUV measurements for PET/CT such as the European Association of Nuclear Medicine (EANM) Research Ltd. (EARL) guidelines. These recommendations, named the EARL2, recently became available for PET/MRI, that also include the higher spatial resolution based EARL2 standard. As pCCA is oftentimes a non-bulky tumour with linear tumour spread along the biliary tree, and consequently likely subject to partial-volume effects SUV measurements on EARL2 PET imaging reconstructions seems most appropriate.

The aim of this study was to assess the added value of FDG-PET/MRI for diagnosis and clinical decision making in patients with suspect pCCA opting for surgical resection. The secondary aim was to measure quantitative PET signal intensity features with the use of EARL2 on the lesion, liver parenchyma, blood pool and correlate these measurements to patient- and tumour characteristics.

## Materials and methods

### Patient Population

Between November 2021 and April 2022, a single-centre, prospective, observational cohort study was conducted (POELH trial; NL9599). Eligible patients were discussed in the multidisciplinary tumour board (MDTB) and included after PET/MRI for work up of suspected resectable pCCA, regardless of eventual histopathology results. The MDTB approved the indication for PET/MRI when diagnosis and staging was uncertain and deemed possible beneficial for clinical management. This study was conducted in accordance with the Helsinki declaration and followed the STROBE guidelines after being approved by the local ethical review committee (MEC-2021-0524). Five patients underwent PET/MRI shortly before the trial formally started and were included retrospectively after providing informed consent. The indications and PET/MRI itself were identical to (the rest of) the study cohort.

### Surgery Work-Up

At the MDTB, comprised of specialized gastroenterologists, oncologists, surgeons, radiologists, and nuclear physicians, all patients with suspected pCCA are discussed to determine the best course of action. All patients underwent a multi-phase CT scan of the liver, including imaging of the full abdomen and thorax, as well as MRI of the liver, including MRCP, for initial assessment. Findings from CT and MRI/MRCP are presented and discussed during the meeting, with particular attention paid to longitudinal tumour extent, vascular invasion and suspect distant metastases. Suspected pCCA is classified according to the Bismuth-Corlette (BC) classification system. Positive regional lymph nodes were not considered a contra-indication for resection, except for liver transplantation. In addition, in the surgery work up an endoscopic biliary drainage with plastic stents is routinely performed in patients. Given that jaundice or cholestasis is a common presenting symptom of pCCA, preoperative biliary drainage is typically performed at our centre when bilirubin levels rise above 70 mmol/L.

### Imaging Protocol for FDG-PET/MRI

Simultaneous PET/MR imaging was performed using a General Electric Healthcare (GE) Signa PET/MR (GE Healthcare, Waukesha, MI, USA). Median time intervals between CT and PET/MRI were 1 week [IQR: 1–2] and between MRI and PET/MRI were 2 weeks [IQR: 0.5–3]. Patient preparation and PET image acquisition was performed in accordance with the European Association of Nuclear Medicine (EANM) Research Ltd. (EARL2) guidelines [19]. Preparation consists of fasting at least 6 h before the PET/MRI. Prior to the scan, serum glucose levels were measured in mmol/L. Each patient underwent whole-body hybrid PET/MRI at 60 (+/-5) minutes after injection of 18 F-FDG (weight 55–100 kg: 0.033 MBq * (kg)^2^, 101–140 kg: 0.025 MBq * (kg)^2^; maximum activity: 500 MBq). Three (55–100 kg) to four minutes (> 100 kg) per bed position; vertex – mid-thigh. Directly following (approximately 120 min after injection), hybrid PET/MRI was acquired for one table position of the upper abdomen, with a full MRI of the liver including contrast- enhanced (Gadolinium- chelates) series, and continuous PET acquisition for the full duration of the MRI liver protocol. All PET data were reconstructed according to the recent EARL2 standard for PET/MR [18]. Standard DIXON based attenuation correction was applied using a four tissue class segmentation (water, fat, lung, air).

SUVmean of the liver was measured by a 100 ml spherical volume of interest (VOI) in the right upper right lobe of the liver (excluding main vascular structures) and SUVmean of the background was measured by a 10 ml spherical VOI in the right heart chamber. SUVpeak, defined by the hottest 1 ml spherical region in the VOI, and SUVmax were measured in spherical VOI’s around the pCCA lesions. The highest SUVpeak and SUVmean were reported for patients with multiple or extended pCCA lesions requiring multiple VOI measurements. The tumour to liver ratio (TLR) and tumour to background ratio (TBR) were calculated by dividing the lesion SUVmax and SUVpeak with respectively the liver SUVmean and background SUVmean. Of note, as pCCA is oftentimes a non-bulky tumour with linear tumour spread along the biliary tree, it is likely that SUVpeak measurements will not be possible and /or unreliable in some patients. All SUV measurements were performed in the Philips VUE PACS viewer (version 12.2).

### Outcome Measures

The primary outcome was the (1) added value for diagnosis and (2) influence on clinical decision making. The added value for final diagnosis was twofold. Firstly, it was based on changes in the TNM staging of PET/MRI compared to CT and MRI/MRCP. Secondly, it was based on combined expert opinion by the reporting radiologist (Dwarkasing) and nuclear physician (Lussanet de la Sabloniere). Influence on clinical decision making was defined as an adjustment in management plan after PET/MRI. This was assessed retrospectively by the research team (Meijering, de Jong, Dwarkasing, Lussanet de la Sabloniere) based on the structured reports of MDTB before and after PET/MRI available in patients digital records. Findings on PET/MRI that showed additional value, but did not affect clinical decision making, such as increasing the suspicion of metastatic disease or benign disease were reported as such, conform two recent studies [20, 21].

Secondary outcomes were associations of SUVmax, TLR and TBR, with patient age, sex, histology, history of Primary Sclerosing Cholangitis (PSC), CA19.9 levels and BC classification. Final diagnosis was based on histopathology proven disease or clinical management with long term follow up, including confirmation by the MDTB.

### Statistical Analysis

For final analysis patients were categorized into two subgroups based on the mean SUVmax of the (suspected) pCCA lesions with high and low SUVmax subgroups with cut-off value equal to the mean value. Significance of patient characteristics with the high and low SUVmax subgroups, as well as TLR and TBR, was evaluated with Fisher’s exact test or Mann-Whitney-U test. Other features were patient age (older or younger than 65 years) and CA19.9-levels (patients with more of less than 37 kU/L). In addition, SUVmax was related to the presence of PSC, final diagnosis and to the BC classification in case of pCCA. All tests were two-sided, and a P-value of less than 0.05 was considered statistically significant. IBM SPSS Statistics (Version 27) was used to perform all statistical analyses.

## Results

### Baseline Characteristics

We included 14 patients (64% male, mean age 62.4 (Standard Deviation (SD) 13.5)). Median BMI was 25.3 [IQR: 23.3–26.7]. Elevated levels of CA19.9 were seen in 9 patients (64%). A history of PSC was present in five patients (36%) (Table 1). Final diagnosis was pCCA in 10 patients (71%), intra-ductal papillary mucinous neoplasm of the bile duct in one patient (7.1%), follicular lymphoma in one patient (7%), IgG4-mediated disease in one patient (7%), and unspecified benign disease in one patient (7%) (Table 2). In 12 patients (86%) final diagnosis was based on patho-histological proof, in two patients (14%) on long term clinical follow-up. In pCCA patients, BC classification based on CT and MRI/MRCP were type 1 (*n* = 3); type 2 (*n* = 3), type 3 A (*n* = 3), type 3B (*n* = 2),and type 4 (*n* = 1) respectively. Median serum glucose levels prior to PET MRI were 6.1 mmol/L [IQR: 5.4–6.2].

**Table 1:** Baseline characteristics of the study population

Patient #	Sex	Age (in years)	History of PSC	BMI	CA19.9 (in kU/L)	Glucose levels (mmol/L)*
1	F	81	-	27.9	183	6.1
2	M	69	-	25.7	348	4.5
3	F	61	+	25.1	111	6.2
4	M	74	-	24.4	1096	5.3
5	M	79	+	20.9	1987	6.2
6	M	69	-	25.3	99	7.2
7	F	68	-	22.6	284	6.9
8	F	64	-	26.9	< 2	5.7
9	M	52	-	26.2	6802	5.8
10	M	61	+	22.4	-	6.3
11	M	56	-	23.4	11	5.1
12	M	50	+	23.2	14	6.1
13	F	28	+	28.4	159	5.3
14	M	61	-	28.6	< 2	6.0

BMI = Body Mass Index, PSC = Primary Sclerosing Cholangitis

* = Prior to PET/MRI

**Table 2:** Cross-sectional imaging, PET/MRI characteristics and follow-up

Patient #	CT		MRI/MRCP		PET/MRI		Stent in situ	SUVmax	TLR	TBR	Diagnostic value	Effect on CDM	Final Diagnosis	Surgery
	TNM^a^	BC	TNM^a^	BC	TNM	BC								TNM
1	T2aN2M0	3a	T2aN2M0	3a	T2aN1M0	3 A	-	3.7	1.09	2.18	PET MRI made lymph nodes suspicious for metastases	- ^a^	pCCA, clinical follow-up	X
2	T2bN1M0	3b	T2bN1M0	3b	T2bN0M0	3B	-	1.8	0.9	1.06	No additional information	-	pCCA, resection (path-proven)	T3N0Mx
3	T2bN2M0	2	T2bN2M0	2	T2bN0M0	2	-	5.6	2.15	2.95	No additional information	-	pCCA, biopsy (path-proven)	X
4	T3N1M0	3a	T3N1M0	3a	T2bN1M0	3 A	PS + PTCD	4.2	1.75	2.21	No additional information	-	Unresectable pCCA, DLS (path-proven)	T4N1Mx
5	T2aN1M0	1	T2aN1M0	1	T2aN1M0	1	-	6.2	2.58	4.77	Increase suspicion of malignancy compared to previous imaging	+	Unresectable pCCA, DLS (path-proven)	TxN2Mx
6	T2aN1M0	3b	T2aN1M0	3b	T2aN1M0	3B	-	6.3	1.91	2.74	No additional information	-	Intraductal high grade dysplasia, resection (path-proven)	Tx/1N0Mx^b^
7	T2aN1M0	3a	T2aN1M0	3a	T2aN0M0	3 A	PS + 2 ucSEMS	2.9	1.26	1.71	No additional information	-	pCCA, resection (path-proven)	T2aN0Mx
8	T2bN1M0	1	T2bN1M0	1	X	X	PS + fcSEMS	5.5	2.2	3.06	PET avidity around the plastic stent most likely due to reactive or inflammatory changes	-	Benign pathology, resection (path-proven)	X
9	T3N1Mx	2	T3N1Mx	2	T2bN0Mx	3 A	2x PS	5.3	2.3	4.08	Enlarged regional LN possible malignant on CT and MRI, not PET avid and not suspicious with PET	- ^c^	pCCA, resection with LN negative (path-proven)	T2aN0Mx
10	T2aN1M0	2	T2aN1M0	2	T2aN0M0	2	PS	6.9	3.45	3.14	No additional information	-	pCCA, biopsy (path-proven)	TxN1Mx
11	T1N0M0	1	T1N0M0	1	T1N0M0	1	-	3.1	1.41	2.07	Increase suspicion of malignancy compared to previous imaging	+	pCCA, resection (path-proven)	T1N1Mx
12	X	X	X	X	X	X	-	5	2.5	3.33	No additional information	-	Follicular lymphoma, resection (path-proven)	X
13	T4N1M0	4	T4N1M0	4	T4N0M0	4	-	5.1	1.59	2.68	Enlarged regional LN possible malignant on CT and MRI, not PET avid and not suspicious with PET	-	pCCA, brush (path-proven)	TxN1Mx
14	X	X	X	X	X	X	-	2.7	1.08	1.59	No additional information	-	IgG4 mediated disease, prednisone treatment with good effect. FU for 9 months	X

IgG4 = Immunoglobulin G 4, SUV = Standardized Uptake Value, TLR = Tumour to Liver Ratio, TBR = Tumour to Blood pool Ratio, DLS = diagnostic laparoscopy, LN = lymph nodes, path-proven = histopathology proven disease, PS = plastic stent, PTCD = percutaneous trans-hepatic catheter drainage, ucSEMS = uncovered self-expanding metal stent, fcSEMS = fully covered self-expanding metal stent, BC = Bismuth-Corlette, CDM = Clinical Decision Making, TNM = Tumour, Node, Metastasis

^a^ Palliative treatment per request of the patient

^b^ IPNB without infiltrative growth

^c^ Regardless of suspicious lymph nodes surgery, therefore no impact on clinical decision making

### TNM Staging, Additional Value and Accuracy

**Fig. 1:**
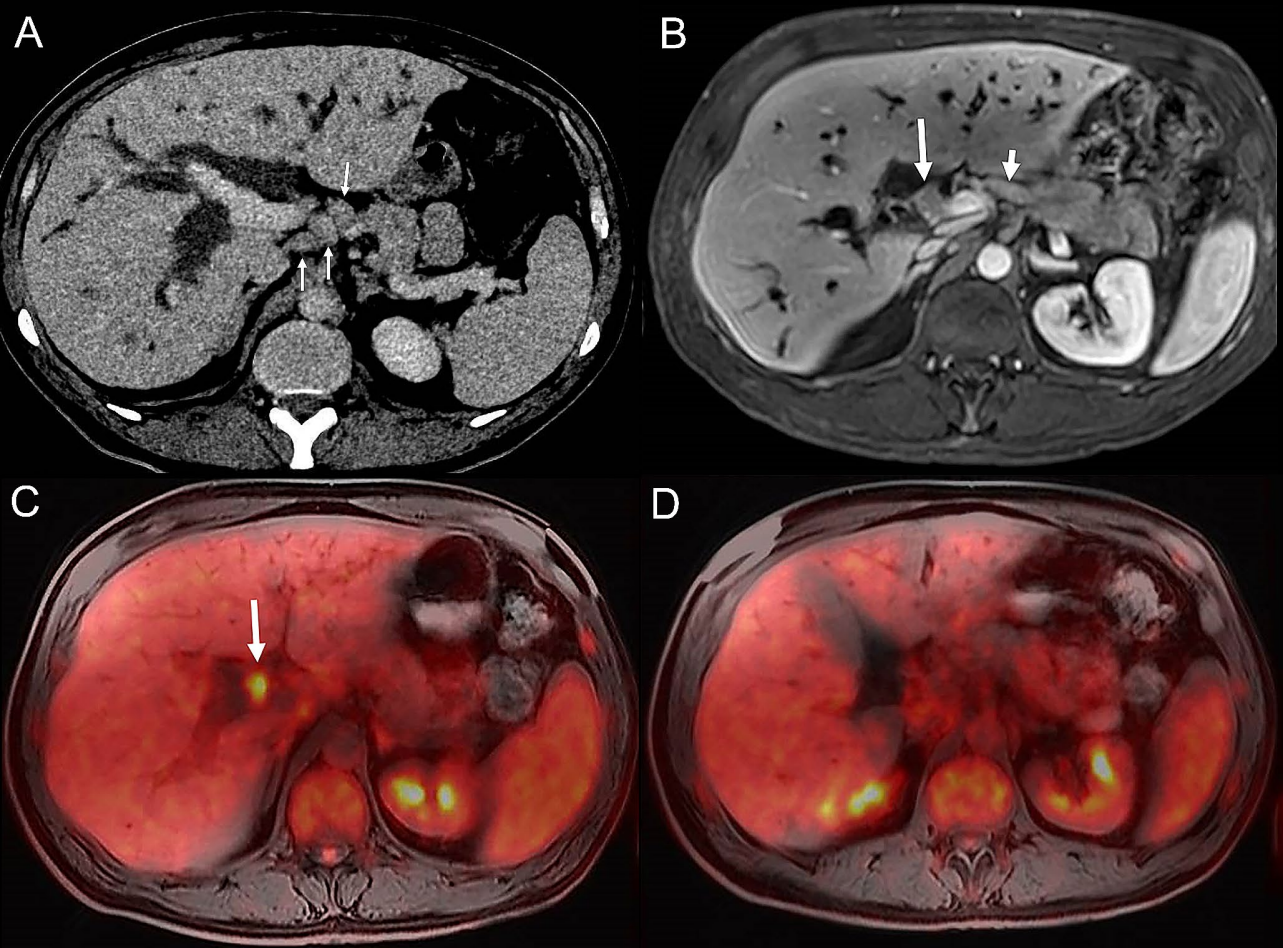
PET/MRI of patient with pathologically confirmed pCCA and negative regional lymph node (#9). An illustrative case of a 52-year-old male with pCCA (histopathology proven). **A**, **B**) Small enhancing tumour mass (**B**, long arrow) is located at the hilum of the biliary tree with dilatation of intrahepatic ducts. Bismuth-Corlette II lesion. In addition, enlarged loco regional lymph nodes (**A**, **B**, small arrow) were seen in the hepatoduodenal ligament. TNM staging based on CT (**A**, axial contras-enhanced CT image) and MRI/MRCP (**B**, axial contrast-enhanced T1-weighted MR image) was T3N1Mx and T2aN1M0 respectively. **C**, **D**) Axial PET/MRI image. The primary tumour demonstrates FDG-avidity (**C**) with no FDG-avidity of the regional lymph nodes (**D**). TNM stage after PET/MRI was T2bN0Mx (N-stage downgraded from N1 to N0 after PET/MRI)

In half of the patients (*n* = 7, 50%), PET/MRI did not present changes to the TNM staging compared to CT and MRI/MRCP. In two cases (14%) the T stage was changed to T2b by PET/MRI instead of T3 on CT (patient #4 & #9). In 7 cases (50%), regional lymph nodes were down staged by PET/MRI due to the following reasons: non FDG-avid LN with a FDG-avid tumour (from N1 to N0, (*n* = 2) and in another 5 patients regional LN identified on CT and MRI/MRCP were not seen on PET/MRI: N2 to N1 (*n* = 1); N2 to N0 (*n* = 1); and N1 to N0 (*n* = 3) (Fig. 1). PET/MRI did not change M- stage, as in all patients no extrahepatic disease was identified by CT, MRI/MRCP and PET/MRI and clinical follow up. In one case (14%) of pCCA BC classification was changed from type 2 to type 3 A (patient #9) by PET/MRI.

In most cases (*n* = 11, 79%) PET/MRI did not present additional diagnostic information to previous CT and MRI/MRCP. The suspicion for primary malignancy changed after PET/MRI in three cases (21%). In one case (7%), benign disease was more probable based on PET/MRI as there was no FDG-avidity of the lesion, no clear mass and no architecture distortion of perihilar anatomy (patient #8). In two cases, high FDG-avidity increased the confidence for malignancy with notable FDG-avidity of a small lesion (patient #5 & #11).

In 11 cases, a surgical staging procedure with or without resection was performed. In two patients, no malignancy was identified and in one patient no pCCA, but a follicular lymphoma was identified. Surgical assessed T stage was available in 5 cases with N stage available in 8 cases. T stage was correctly identified on CT (*n* = 2, 40%); MRI/MRCP (*n* = 3, 60%); and PET/MRI (*n* = 2, 40%) respectively. N stage was correctly identified on CT, MRI/MRCP, and PET/MRI in 4 cases (50%), respectively.

### Influence on Clinical Decision Making

PET/MRI affected clinical decision making in two patients (14%). In these two patients (patient #5 & #11), PET/MRI increased the suspicion of primary malignancy solely based on high tumour FDG avidity compared to the liver, which resulted in a decision to perform surgery, instead of possible further invasive diagnostic procedures or conservative management. Both patients had surgically confirmed pCCA. In the remaining 12 patients (85.7%), PET/MRI did not affect clinical decision making (Table 2*)*.

### Measurement of SUVmax, Correlated with Patient Characteristics

The mean SUVmax value for the PET/MRI of the liver was 4.59 [95%CI: 3.81–5.37]. High and low SUVmax subgroups were created relative to the mean SUVmax value of the liver (4.59); both consisted of 7 patients each. Correlations of SUVmax with baseline clinical characteristics was evaluated (Table 3). In most patients (*n* = 13) one spherical VOI’s around the perihilar lesion was applied for SUVmax measurement. One patient had measurements with multiple spherical VOIs of the lesion due to the extended shape of the tumour.

**Table 3:** Correlation of baseline characteristics and SUVmax (Fisher’s exact test)

Characteristic	High SUVmax (> 4.59)	Low SUVmax (< 4.59)	Total	*P*-value
Age in years				
- ≥ 65	2	4	6	0.28
- < 65	6	2	8	
Sex				
- Male	5	4	9	0.99
- Female	3	2	5	
Histology^a^				
- Malignant	5	5	6	1.00
- Benign	1	1	6	
PSC diagnosis				
- Yes	5	0	5	0.03
- No	3	6	9	
CA19.9				
- ≥ 37 kU/L	6	3	9	0.99
- < 37 kU/L	2	2	4	
BC-type				
- I-IIIA	5	4	9	0.99
- IIIB-IV	2	1	3	

PSC = Primary Sclerosing Cholangitis, SUV = Standardized Uptake Value, BC = Bismuth-Corlette

^*a*^ Excluding the patient with lymphoma and IPNB

**Fig. 2:**
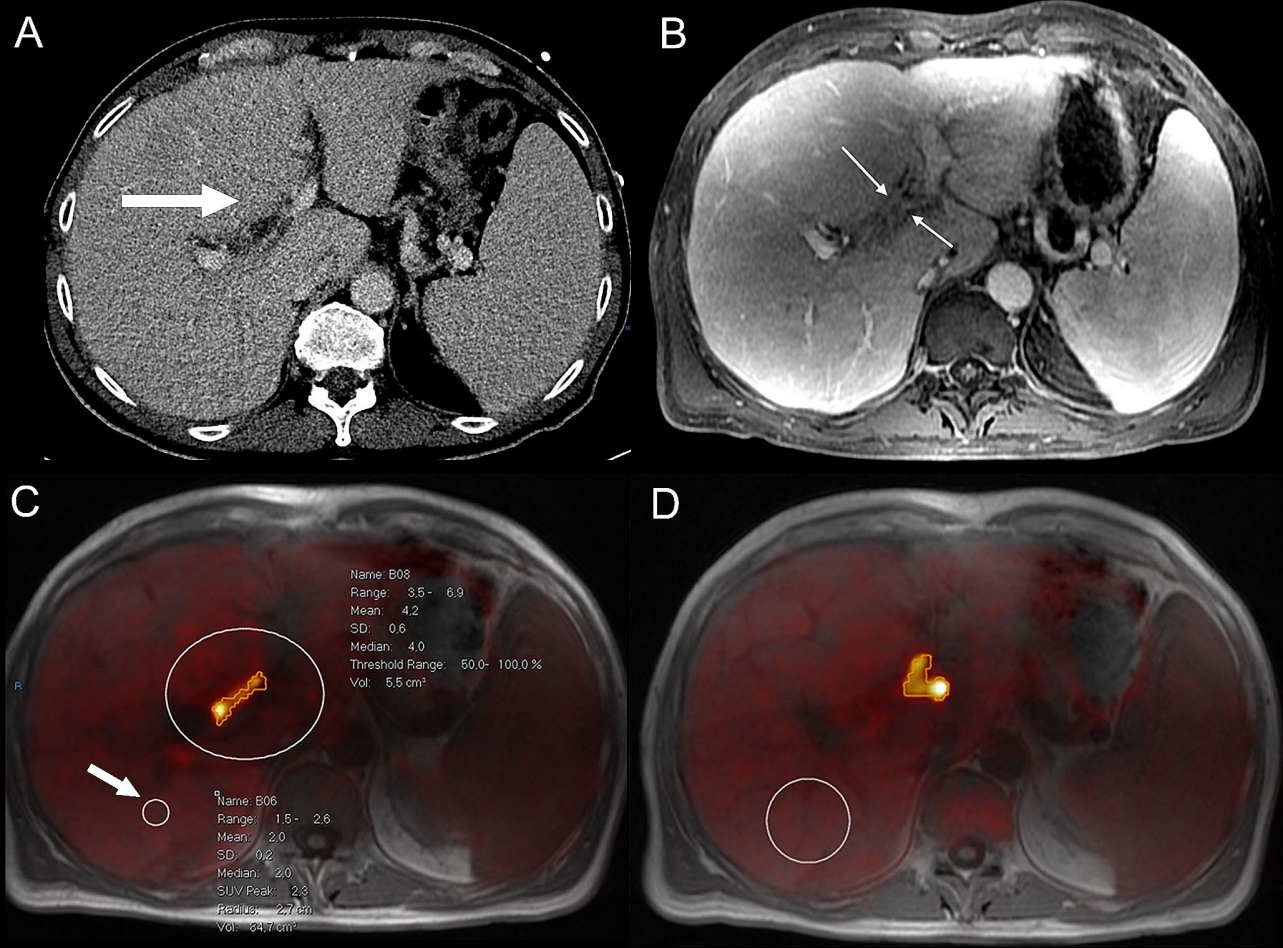
PET/MRI of patient with pathologically confirmed pCCA (#10). The PET/MRI shows a pCCA, BC type IIIB. Measurements show a SUVmax of 6.9, TLR of 3.45 and TBR of 3.14. An illustrative case of a 61-year-old male with pCCA (histopathology proven). The tumour is located in the perihilar region (**A**, arrow) and extending past the first branching of the left hepatic main duct (**B**, arrows), classified as a Bismuth-Corlette III-B tumour. A) With CT no obvious lesion is discernible in the perihilar region. **B**) On contrast-enhanced MRI the tumour can be seen as subtle irregular thickened bile duct wall with no clear tumour mass (arrows). **C**, **D**) Clear FDG-avid lesion with limited tumour volume (segmented), including ROI placement (**C**, arrow) to measure liver background activity and tumour activity

**Fig. 3:**
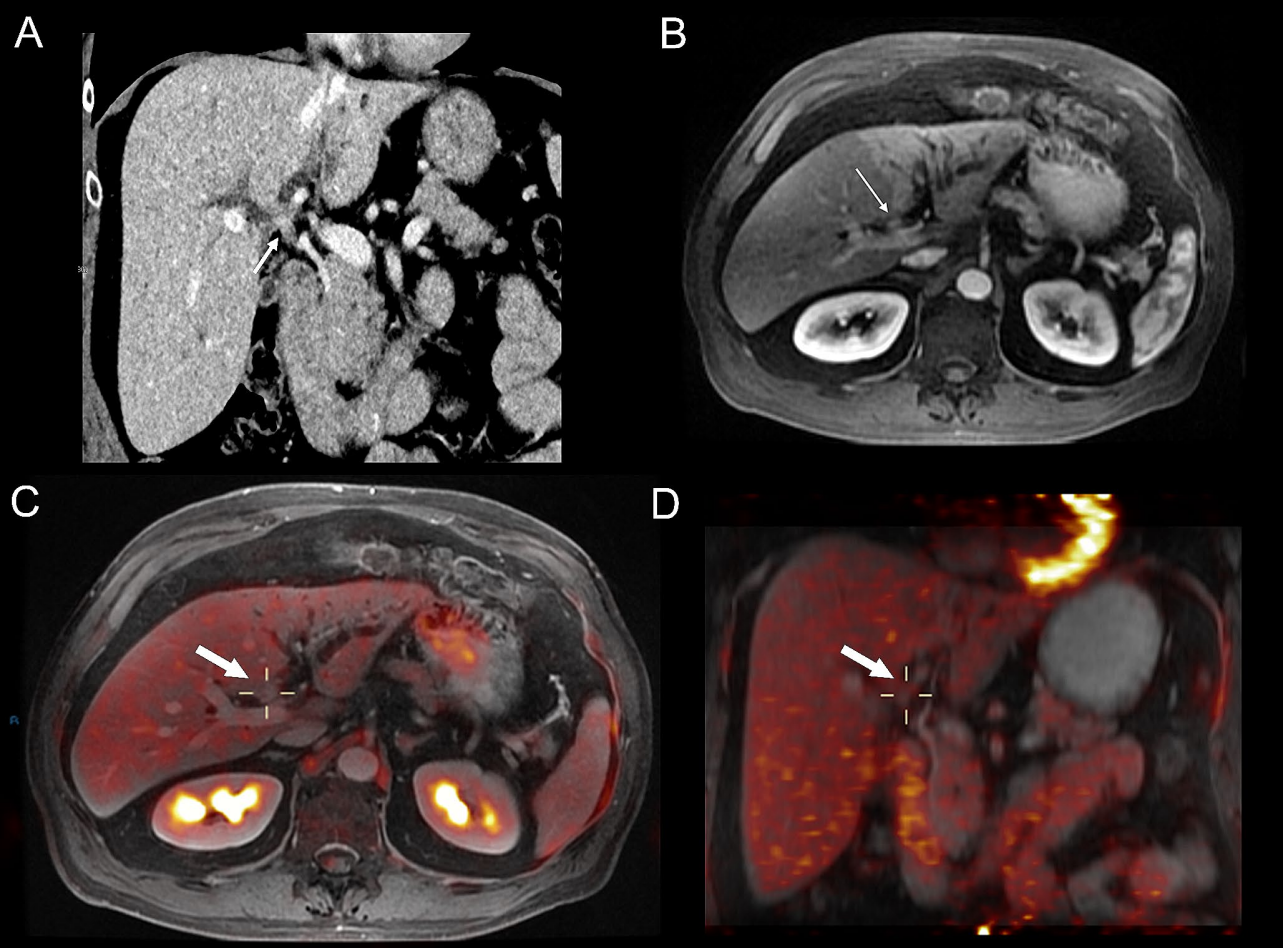
PET/MRI of patient with pathologically confirmed pCCA (#2). An illustrative case of a 69-year-old male with pCCA (histopathology proven). Small tumour mass is located at the hilum of the biliary tree with marked dilatation of the left hepatic ducts, including atrophy of the left liver lobe, Bismuth-Corlette II lesion. **A**) Coronal CT iage with a small obstructive tumour in the biliary hilum (arrow), including proximal intrahepatic bile duct dilatation. **B**) On axial contrast-enhanced MRI the lesion can be appreciated as a small localized enhancing tumour (arrow). **C**, **D**) Axial and coronoal PET/MRI images show a small pCCA, BC type I with low PET avidity (arrows), including relatively low SUVmax (1.8), TLR (0.9), and TBR (1.06)

**Fig. 4:**
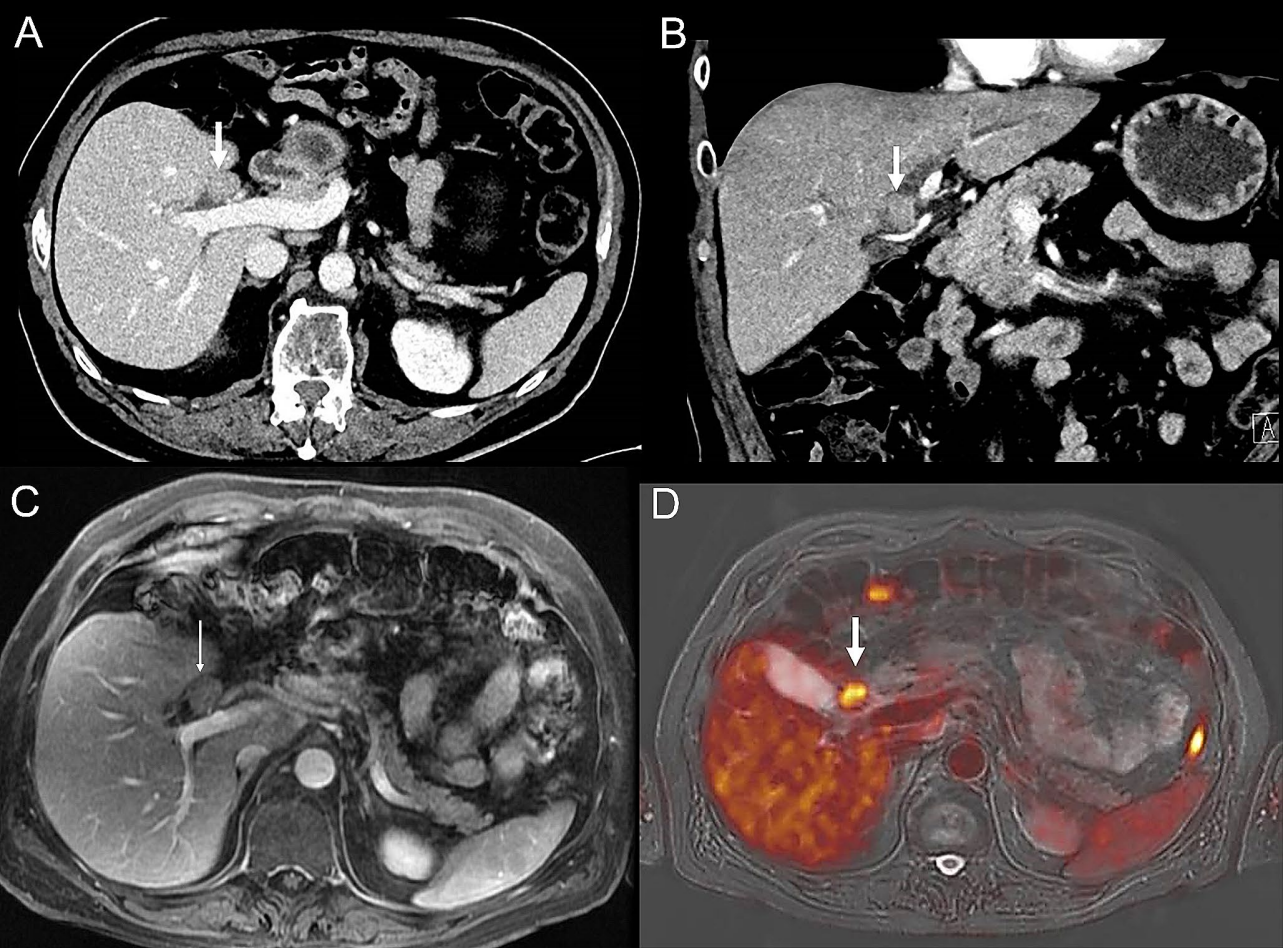
PET/MRI of patient with pathologically confirmed intra-ductal tubulopapillary neoplasm with dysplastic cells (#6). An illustrative case of a 69-year-old male with intra-ductal tubulopapillary neoplasm with dysplastic cells (histopathology proven), no infiltrative growth. **A**,**B**) CT images demonstrate a well-defined intra-ductal obstructive mass lesion with contrast enhancement (arrows) similar to the surrounding liver parenchyma. **C**) Axial Contrast-enhanced T1-weighted MRI image with no additional findings compared to CT (**A**, **B**), **D**) Axial PET/MRI shows an obvious FDG-avid lesion (arrow)

A significant correlation was found for high SUVmax and history of PSC (*p* = 0.03). No significant correlation was found between SUVmax and age (*p* = 0.28), sex (*p* = 0.99), CA19.9 levels (*p* = 0.99), histopathology proven cholangiocarcinoma (*p* = 0.99), and BC type pCCA (*p* = 0.99) (Table 3*)*. SUVmax threshold that could differentiate between benign and malignant lesions was not achievable mainly because of the limited number of non-CCA cases. Cases with pCCA (*n* = 10) showed either relatively high SUVmax values (*n* = 5) (Fig. 2) or low SUVmax values (*n* = 5) (Fig. 3). Furthermore, benign lesions demonstrated both low (*n* = 1) and high (*n* = 1) SUVmax values (Fig. 4).

### Measurements of TLR and TBR, Correlated with Patient Characteristics

The mean TLR was 1.87 [95%CI: 1.51–2.23]; the mean TBR was 2.63 [95%CI: 2.13–3.14]. There were no significant associations between TLR and final diagnosis (*p* = 0.67), nor between TBR and final diagnosis (*p* = 0.89). No significant correlation was found when analysing TLR as dichotomous outcomes with cut-off value of 1.3.

## Discussion

Our study found a notable disparity in TNM staging, observed in 50% of patients, between hybrid 18-FDG-PET/MRI and conventional cross-sectional imaging using CT and MRI/MRCP. The additional diagnostic benefit provided by PET/MRI amounted to 21%, and PET/MRI influenced clinical management in merely 14% of patients with suspected pCCA eligible for surgery. Furthermore, with PET signal intensity measurements we found a significant correlation between a high lesion SUVmax and history of PSC (*p* = 0.03).

Among the eleven patients that underwent surgical exploration or resection, PET/MRI accurately altered the N stage in three patients. Conversely, PET/MRI incorrectly estimated the T stage in two patients and incorrectly changed the N stage from N1 to N0 in two patients. These findings align with a recent study of Obman et al., which reported that FDG-PET/MRI correctly adjusted the cTNM stage in 22% of patients with various hepatobiliary malignancies [14]. Regarding changes in the N stage, a potential explanation may be that prominent lymph nodes are more distinctly visualized using dedicated CT and MRI/MRCP scans with contrast. In PET/MRI, these lymph nodes are only identified when they demonstrate avid activity on the PET image, and correlated with the MRI image. Normal lymph nodes lacking PET avidity are therefore not visible on regular PET/MRI, while these are clearly discernible on dedicated CT and MRI/MRCP. A potential advantage of PET/MRI compared to PET/CT has been described by Catalano et al., which found PET/MRI to characterize lymphadenopathy more accurately then same-day PET/CT [20]. Correct identification of lymph nodes on cross-sectional imaging may guide preoperative endoscopic ultrasound to target specific suspicious lymph nodes, which can preclude unnecessary surgical exploration [22–25].

Correlation of our findings to recent literature is challenging, as other studies primarily focused on different types of hepatobiliary cancer [14, 20, 21, 26, 27]. For instance, Obman et al. reported a change in management in 11% of patients [14]. In a study involving patients with cancer (excluding pCCA) who underwent same-day PET/CT and PET/MRI, PET/MRI influenced clinical management in 18% of patients compared to PET/CT [20]. The criteria for influence on clinical management in these papers was similar as applied in our study. In another study involving 263 patients, including three patients with cholangiocarcinoma, PET/MRI influenced management in 8%, although not in the cholangiocarcinoma patients [21]. A recent study of fifteen patients with hepatocellular carcinoma showed no impact of PET/MRI on clinical decision making [28]. Our study is the first exclusively focused on pCCA, where the role of PET/MRI was evaluated. Clinical decision making was affected in two patients (14%), both had an increased suspicion of malignancy due to high SUVmax of the pCCA lesions.

Previous studies on PET/MRI using 18-FDG have suggested that high values of SUVmax, TLR, and TBR would favour malignant tumours [29, 30]. Utsonomiya et al. found a TLR > 1.3 measured on PET/CT to be an independent predictor of malignancy for pancreatic intra-ductal papillary mucinous neoplasms [29]. We were unable to reproduce these findings with pCCA both as a continuous variable and as a dichotomous variable using the same cut-off value. Two patients with non-malignant disease in our study, specifically with high-grade dysplasia and unspecific benign disease who underwent resection, had high TLR values. In clinical practice, it is generally assumed that malignant lesions will display notable FDG-avidity with high TLR value. Our observation does not support these assumptions, although our findings are limited by the small number of included patients with benign disease. In addition, all cases of pCCA in underlying PSC livers (*n* = 5) demonstrated high SUVmax values. This was a significant finding and may be an important imaging marker for pCCA in PSC patients. Validation of this finding is warranted in larger study populations.

Tomimaru et al. investigated the use of SUVmax values in differentiating malignant from benign intraductal papillary mucinous neoplasms (IPMNs) in 29 patients [31]. The results showed that SUVmax values were significantly higher in malignant IPMNs compared to benign IPMNs and were positively correlated with the histopathological types of IPMN. The best diagnostic accuracy was achieved by using a SUVmax cut off value of 2.5. Combining this with the detection of a mural nodule on CT offered the most effective diagnosis of malignant IPMN. In our study we could not find a trend to differentiate malignancy from benign disease based on SUVmax, TLR, and TBR. On top of that, we would like to emphasize that a cut-off value for high or low SUVmax that could be indicative for differentiating malignancy from benign disease was not achieved in our study. We believe, based on our results, that SUVmax should be interpreted as a continuous variable and future (large) studies should reveal the true value of SUVmax measurements for diagnosis and treatment outcome. Consequently, one cannot rely solely on these quantitative parameters for diagnosis and clinical management of obstructive perihilar biliary lesions. Clinical work up, expert reading of baseline imaging (CT scan and MRI/MRCP) and multidisciplinary evaluation in expertise centres is recommended for these patients. FDG-PET imaging of pCCA is challenging because of the non-bulky, linear tumour spread along the biliary tree that may negatively affect visible PET avidity owing to partial volume effects and motion artefacts. For this reason, we welcome efforts to standardize reproducibility of PET imaging features such as EARL2 in SUV measurement as was implemented in our study. Our results show that PET/MRI may increase the confidence level for malignancy which may speed up the clinical decision to perform surgery. The indication for PET/MRI should therefore be considered on a case-by-case basis. PET/MRI may be valuable to demonstrate the full extent of lesions (Fig. 2), assist in characterization of enlarged lymph nodes and detect distant metastases that were not apparent on previous CT and MRI/MRCP. Another advantage of PET/MRI compared to PET/CT is higher sensitivity for bony and hepatic metastases [20]. In our study however, we had no cases with distant metastases.

Cholangiocarcinoma is histologically an adenocarcinoma and typically shows elevated FDG uptake. There are two important limitations with FDG uptake in case of pCCA: (1) FDG-avidity may be decreased, due to abundance of fibrotic stroma in pCCA and (2) FDG tracer enables detection of glycolysis, present in the whole body, and increased in areas of infection and inflammation which is common along the biliary tree after stent placement for pCCA. It is therefore recommended to perform PET/MRI before biliary stent placement. Since 2019, fibroblast activation protein inhibitor (FAPI) is being investigated in the setting of cholangiocarcinoma as a new PET-tracer [32]. Cancer-associated fibroblasts show a high expression of fibroblast activation protein, which can be better demonstrated by using FAPI-tracer, while expression levels in normal human tissue are generally very low [33]. A recent systematic review shows the potential that FAPI PET/MRI holds for pCCA patients [34]. Future studies with FAPI PET/MRI may show higher specificity for diagnosis and staging of pCCA.

To the best of our knowledge, this is the first prospective study on the added value of PET/MRI in presumed resectable pCCA patients exclusively. Limitations include the small number of patients in this study. Although our centre is one of the largest referral centres for cholangiocarcinoma management in the Netherlands, the number of patients presenting annually with potentially resectable pCCA is low. Unfortunately due to the small number of patients we were unable to perform additional analyses, such as correlation between PET/MRI features and clinical data. Another limitation might be selection bias, which may have influenced our results. Patients were included when pCCA was suggested on clinical grounds. Still three patients were included with no malignancy. We consider this finding in line with real world data. It is a well-known fact that benign biliary obstructive lesions, especially inflammatory disease, may mimic pCCA [35]. Another limitation might be that the influence on clinical decision making was not clearly defined in advance but was interpreted retrospectively. Our patients have structured documentation on the MDTB decision and recommendation for clinical management in their electronic medical files. In addition, the influence on clinical decision making was based on group discussions with four members of our research team on MDTB reports before and after PET/MRI.

## Conclusions

In summary, 18 F-FDG-PET/MRI has limited value for diagnosis of pCCA and influenced clinical decision making in 14% of pCCA patients opting for surgery. The indication for 18 F-FDG-PET/MRI should therefore be considered selectively on a case-by-case basis. Prospective multicentre trials on hybrid PET/MRI with more sensitive tracers (e.g. FAPI) and standardized evaluation methods (e.g. EARL2 based SUV) in the setting of pCCA are warranted.

## Data Availability

The datasets analysed during the current study are available from the corresponding author upon reasonable request.

## Abbreviations

FDG: 18 F-fluro-deoxy-glucose
PET: Positron Emission Tomography
MRI: Magnetic Resonance Imaging
MRCP: Magnetic Resonance Cholangio-Pancreatography
CT: Computed Tomography
pCCA: Perihilar Cholangiocarcinoma
SUV: Standardized Uptake Value
PSC: Primary Sclerosing Cholangitis
EANM: European Association of Nuclear Medicine
EARL: EANM Research Ltd
TNM: Tumour, Node, Metastasis
STROBE: Strengthening the Reporting of Observational Studies in Epidemiology
MDTB: Multidisciplinary Tumour board
BC: Bismuth-Corlette
VOI: Volume of interest
TLR: Tumour to liver ratio
TBR: Tumour to background ratio
SD: Standard Deviation
